# Primary outcome measures used in interventional trials for ankle fractures: a systematic review

**DOI:** 10.1186/s12891-019-2770-2

**Published:** 2019-08-28

**Authors:** Rebecca McKeown, Abdul-Rasheed Rabiu, David R. Ellard, Rebecca S. Kearney

**Affiliations:** 1 Warwick Medical School, Warwick Clinical Trials Unit, University of Warwick, Clinical Sciences Research Laboratories, University Hospitals Coventry and Warwickshire, Clifford Bridge Road, Coventry, CV2 2DX UK; 2 Trauma and Orthopaedics Department, King’s College Hospital, Denmark Hill, London, SE5 9RS UK

**Keywords:** Ankle, Ankle injuries, Ankle fractures, Outcome measures, Randomised controlled trials, Patient reported outcome measures

## Abstract

**Background:**

Ankle fractures cause considerable pain, loss of function and healthcare resource use. High quality randomised controlled trials are required to evaluate the optimal management protocols for ankle fracture. However, there is debate regarding the most appropriate outcome measure to use when assessing patients with ankle fractures. The aim of this systematic review is to identify and summarise primary outcome measure use in clinical trials of non-pharmacological interventions for adults with an ankle fracture.

**Methods:**

We performed comprehensive searches of the Medline, Embase, CINAHL, AMED and Cochrane CENTRAL databases, as well as ISRCTN and ClinicalTrials.gov online clinical trial registries on 19/06/2019 with no date limits applied. The titles and abstracts were initially screened to identify randomised or quasi-randomised clinical trials of non-pharmacological interventions for ankle fracture in adults. Two authors independently screened the full text of any articles which could potentially be eligible. Descriptive statistics we used to summarise the outcome measures collected in these articles including an assessment of trends over time. Secondary analysis included a descriptive summary of the multi-item patient reported outcome measures used in this study type.

**Results:**

The searches returned a total of 3380 records. Following application of the eligibility criteria, 121 records were eligible for inclusion in this review. The most frequently collected primary outcome measures in this type of publication was the Olerud Molander Ankle Score, followed by radiographic and range of movement assessments. There was a total of 28 different outcome measures collected and five different multi-item, patient reported outcome measures collected as the primary outcome measure. There was a sequential increase in the number of this type of study published per decade since the 1980’s.

**Conclusion:**

This review demonstrates the wide range of measurement methods used to assess outcome in adults with an ankle fracture. Future research should focus on establishing the validity and reliability of the outcome measures used in this patient population. Formulation of a consensus based core outcome set for adults with an ankle fracture would be advantageous for ensuring homogeneity across studies in order to meta-analyse trial results.

**Electronic supplementary material:**

The online version of this article (10.1186/s12891-019-2770-2) contains supplementary material, which is available to authorized users.

## Background

Ankle fractures are a significant injury which cause pain, reduced function and have a substantial impact on activities of daily living for the individual [1]. Epidemiological studies show that the injury exhibits a bimodal distribution, with ankle fractures sustained in high energy falls more likely to affect a young male population and a those sustained in low energy falls usually affecting older females [2]. Ankle fractures present substantial economic burden to society and healthcare services [3] and their prevalence is increasing due to the ageing population [4, 5].

In light of the increased incidence of ankle fractures, further high quality randomised controlled trials (RCTs) to determine optimal management strategies of this injury are required, as recommended by a Cochrane review of rehabilitation for ankle fractures completed in 2012 [6]. The results of RCTs can be combined in systematic reviews and meta-analyses, widely considered in evidence based medicine as the highest level of evidence [7]. However, meta-analyses are often confounded by a lack of homogeneity of methods and primary outcome measures used.

The choice and use of appropriate primary outcome measures is of utmost importance in the design and conduct of clinical trials and the use of invalid or unreliable outcome measures is a waste of resources and can be regarded as unethical [8]. Traditionally, outcome measures used in orthopaedic clinical practice consisted of clinically based measurements such as radiological findings or assessments of range of movement [9]. Over the past decade, there has been an increasing trend towards the use of patient reported outcome measures (PROMs) in clinical practice and research, in a move towards value-based healthcare delivery [9, 10]. However, debate continues regarding the most appropriate outcome measure to use for adults recovering from ankle fracture [11–13]. The aim of this exploratory systematic review is to identify the primary outcome measures used in published and registered clinical trials of non-pharmacological interventions for ankle fractures in adults. Trends of outcome measure use over time and identification of multi-item patient reported outcome measures used in these types of studies will also be analysed. This systematic review will be conducted in accordance with the Preferred Reporting Items for Systematic Reviews and Meta-analyses (PRISMA) checklist (Additional file 1: PRISMA Checklist) [14] and will follow the Cochrane methodology for systematic reviews [15].

## Methods

### Literature search

A comprehensive search of the Medline, Embase, CINAHL, AMED and Cochrane CENTRAL Trials databases and the ISRCTN and ClinicalTrials.gov registries was completed on 19/06/2019 with no date limits applied. The review was not prospectively registered as it did not meet the criteria for registration with PROSPERO. The search strategies used in this review are found in the additional files (Additional file 2: search strategy).

### Eligibility criteria

Articles and records included were RCTs or quasi-RCTs of two or more non-pharmacological interventions for the management of ankle fractures in an adult population. Quasi-RCTs were included because this review summaries choice of outcome measure rather than summating and evaluating treatment effects, therefore the use of gold-standard randomisation methods was of lesser importance here. Adult was defined as skeletally mature participants and studies evaluating paediatric fractures were excluded due to the differing nature of these injuries [16]. Articles evaluating interventions for Pilon fracture types were excluded due to the differing mechanism of injury and increased risk of associated complications in comparison to malleolar ankle fractures [17, 18]. Studies that included a mixed population of lower limb injuries were excluded, as were longer term follow up studies with collection of no new outcome measures (only initial paper was included). Published protocol papers or registry records of articles which have subsequently been published and appear in the database search results were excluded to avoid duplication. Any records that did not provide sufficient levels of information of the outcome measures being collected were excluded.

### Screening and data extraction

Results from the database searches were exported to EndNote X5 (Thompson Reuters) for review. Duplicates were removed and the eligibility criteria were applied by the lead author through a review of the titles and abstracts. Based on this initial screening, articles were excluded if it was clear from the title and abstract that it did not meet the inclusion criteria. If eligibility was unclear, the lead author retrieved the full text for review. Results from the ISRCTN and ClinicalTrials.gov registries were reviewed through their respective webpages using an internet browser (Google Chrome) and the eligibility criteria were applied.

A second reviewer (AR) independently repeated the application of the eligibility criteria on all papers and registry records for which the full text was retrieved and applied the same criteria, to reduce error and ensure no ineligible records had been included. Where discrepancies occurred, RM and AR discussed and reached consensus on article inclusion. Where consensus could not be reached, a third reviewer (RSK) was consulted for a final decision. Caution was taken throughout the selection process to ensure that articles with a registry entry and a subsequent publication were only included once.

The lead author recorded study details, including authors, title year of publication, journal and outcomes collected in a data extraction spreadsheet using Microsoft Excel (Microsoft 2011). Where authors did not explicitly declare the primary outcome measure (or if multiple were stated), the outcome measure on which the sample size was calculated was used as the primary outcome. Where articles did not state this, the first outcome measure declared in the abstract was regarded as the primary outcome measure. In any cases where this did not apply, the first outcome measure mentioned in the full text of the article was regarded as the primary outcome measure. This method has been used in previous systematic reviews of this nature [19].

### Analysis

We used descriptive statistics to explore what primary outcome measure were used in this type of interventional trial. Secondary analysis explored the number of trials published over time and the temporal trends of the most frequently used outcome measures. An analysis of the multi-item, patient reported outcome measures collected as the primary outcome measures in these studies was also completed.

For the purposes of analysis, radiographic outcome measures collected, such as X-Ray, Computed Tomography (CT) scans and other will be grouped into one category called radiographic assessment. Measurements of swelling, strength and range of movement assessments (ROM) may be performed in a variety of ways between studies, but these will be grouped together as one category (i.e. swelling, strength and ROM) regardless of the specific method of measurement.

## Results

**Fig. 1:**
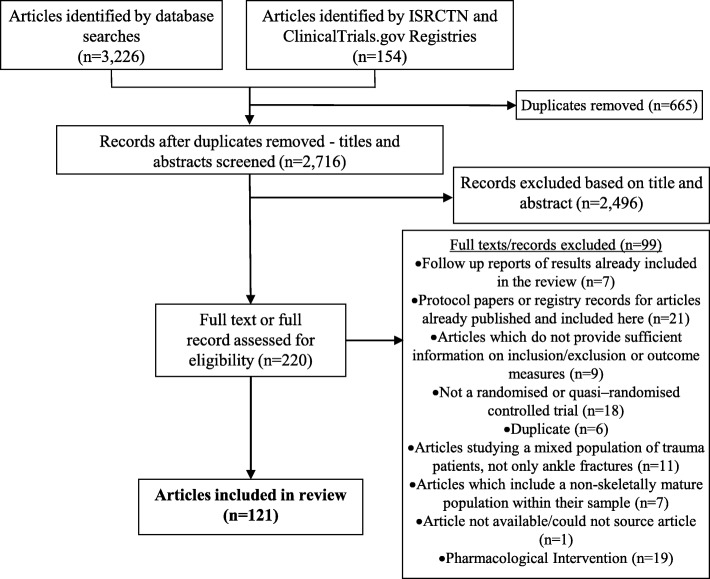
PRISMA Diagram

The searches returned a total of 3380 records, of which 3226 were published articles form database searches and 154 records from registry databases. Following removal of duplicates and application of the eligibility criteria, 121 records were eligible for inclusion in this review. The PRISMA diagram in Fig. 1 shows the reasons for exclusion of articles and records.

The 121 records included were by authors in 21 different countries with a total patient population of 11,662 participants (including planned populations of protocol papers and registry records). The additional files contain the data extraction table including details of the records and the outcome measures collected (Additional file 3: Data Extraction Table). 67 articles/records specified their primary outcome measure (or demonstrated a sample size calculation based upon the minimally clinically important difference of a specified outcome measure) and 54 did not.

Table 1 shows the number of studies in this research area has risen sequentially by decade with a total of 50 articles published this decade so far, compared to eight in the 1980’s.

**Table 1:** Published studies and records by decade

Decade	1980’s	1990’s	2000’s	2010’s	Registered
Number of articles published	8	16	22	50	25

**Fig. 2:**
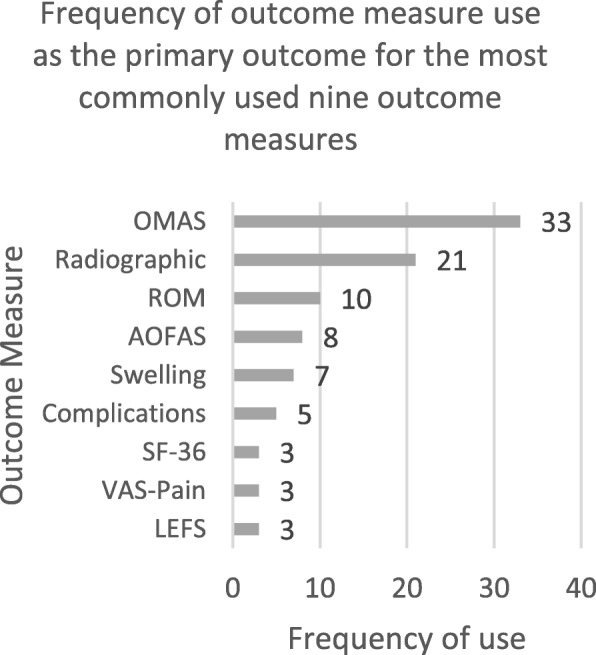
Bar chart showing frequency of use of most commonly used eight primary outcome measures in included studies

As Fig. 2 shows, the most frequently collected outcome measure was the Olerud Molander Ankle Score (OMAS), which was collected as the primary outcome measure in 33 of the 121 records included here. Radiographic assessments followed this, which were analysed 21 times and measurements of ankle ROM were analysed in ten trials as the primary outcome measure. Researchers used the American Orthopaedic Foot and Ankle Society Ankle Hind-Foot Scale (AOFAS) in eight trials and measurements of ankle swelling seven times as the primary outcome measure of the articles included here. Complications or adverse events were collected as the primary outcome measure five times. The Lower Extremity Functional Scale (LEFS), the 36-item Short-Form Survey (SF-36) and Visual Analogue Scale for pain (VAS-Pain) were all collected three times respectively as the primary outcome measure in the included studies.

The remaining outcome measures (not included in Fig. 2) were the Manchester Oxford Foot Questionnaire (MOXFQ), American Academy of Orthopaedic Surgeons Foot and Ankle Outcomes Score (AAOS), Mazur score, blood biochemistry analysis, strength tests, clinical exam, duration of hospitalisation and duration of surgery; these measures were all collected twice as the primary outcome measure. The Kaikkonen score, deep vein thrombosis (DVT) rates, the Baird Score, Weber Score, a Likert scale of satisfaction with treatment, gait analysis, time to surgery, return to work, intraoperative assessment, further procedures and feasibility of recruitment were all collected once as a primary outcome measure in the records included here. There were 28 different outcome measures collected as the primary outcome measure in this type of study.

**Fig. 3:**
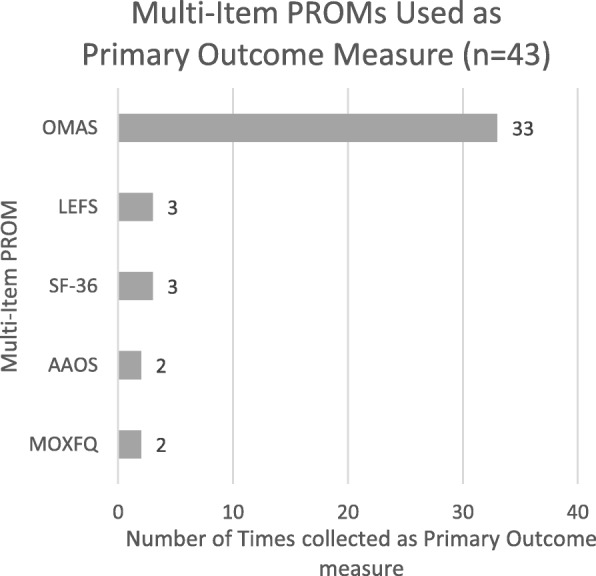
Bar chart showing frequency of multi-item PROM use in included studies

Figure 3 shows the multi-item PROM use in these articles; 43 articles collected a PROM as the primary outcome measure and there were five different multi-item PROMs collected here as the primary outcome measure. As previously discussed, the OMAS, a nine-item, ankle fracture specific PROM [20], was the most frequently collected. This is followed by the LEFS, a twenty-item score to measure function for individuals with musculoskeletal conditions of the lower limb [21]. The SF-36, a 36-item health related quality of life measure [22], was collected three times here in the included records. The MOXFQ [23] and the AAOS [24] were collected twice each; they are 16-item and 25-item (respectively) region specific PROMs designed for the assessment of individuals with conditions affecting the foot or ankle.

**Fig. 4:**
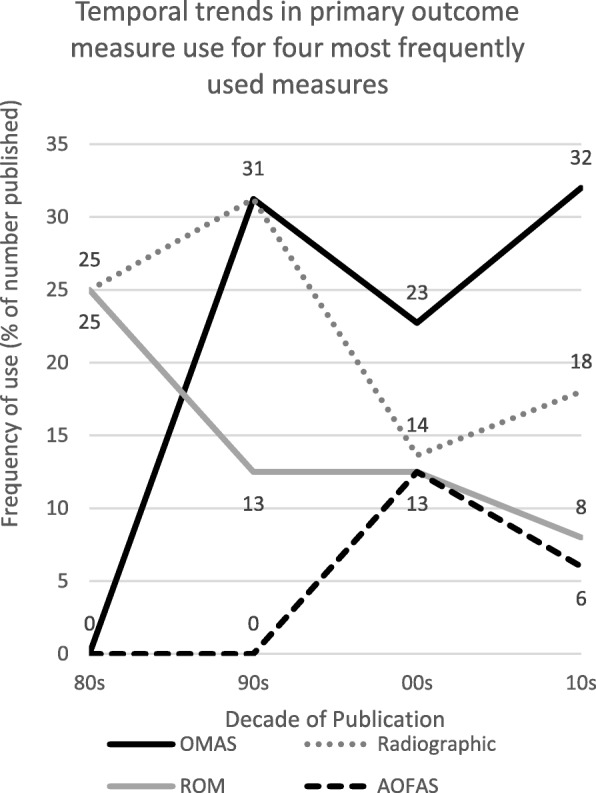
Graph showing temporal trends of most commonly used five outcome measures

Figure 4 shows the temporal trends of the four most frequently used primary outcome measures in trials included in this review as a percentage of the articles published in the respective decade. As demonstrated, the OMAS has been steadily increasing in use in the studies included here since its development, with 14 articles collecting this outcome measure out of 50 (32%) in this decade so far. Radiographic measures have similarly been increasing in use, with a slight reduction in use between 2000 and 2010; radiographic assessments were collected nine times out of 50 articles (18%) as the primary outcome measure in this decade. The AOFAS is a nine-item measure which combines patient reported items with clinician assessed measurements such an ROM, gait and joint alignment. This measure started to be used between 2000 and 2010 and has been steadily used since, between two and three times per decade. Assessments of ROM has been declining as a percentage of published articles since the 1980’s, being used twice as the primary outcome measure in the 80’s (25%) and four times out of 50 articles in the current decade (8%).

## Discussion

This review highlights frequency of use of particular outcome measures over others in clinical trials of non-pharmacological interventions for ankle fracture, most notably the OMAS. Despite some evidence for the validity and reliability of the OMAS [25, 26], there is debate amongst clinicians and researchers regarding its suitability for use, particularly with regard to the lack of a formal development process and a systematic review highlighted concerns with this in particular [11]. The research group recommended the use of a newer PROM, developed in 2014 specifically for this injury, known as the Ankle Fracture Outcome of Rehabilitation Measure (A-FORM) [27]. However, the researchers noted that there was little post-formulation validity and reliability testing on this outcome measure and results here show that this it has yet to be collected as a primary outcome measure in this topic. Whilst researchers are still using the OMAS frequently in this research area, further research into the validity and reliability of this very widely used questionnaire is warranted.

The evidence for use of radiological findings as an outcome measure in ankle fracture is mixed, with some researchers proposing that measurements such as medial clear space and tibiofibular overlap could be predictors of outcome in relation to patient reported outcome [28, 29]. However, other researchers have reported weak association between radiographic measurements and functional outcome measures in individuals with upper limb trauma [30] and elective foot surgery [31]. Whilst the association between radiographic outcome and patient perceived outcome has not yet been evaluated in individuals with ankle fracture, some researchers have questioned the value in performing routine follow-up radiographs for this injury, as they rarely initiate a change in management plan in the absence of patient reported symptoms [32]. This article demonstrates that radiographic outcome is still widely collected as a primary measure in this type of clinical trial without sufficient evidence that it has any association with patient perceived outcome in individuals with an ankle fracture.

This review demonstrates that the AOFAS is still primarily analysed in this type of article, despite several articles providing evidence of insufficient measurement properties of this measure [33, 34]. In 2011, the AOFAS Research Committee recommended that this outcome measure should no longer be used, secondary to robust evidence for a lack of validity and reliability [35]. Despite this recommendation, there are still registered but unpublished articles using AOFAS as their primary outcome measure in clinical effectiveness trials for ankle fractures. Whilst we acknowledge that implementation of research takes time, it’s important that research teams thoroughly examine appropriate outcome measures prior to the formulation of the trial protocol and registration.

This is the first systematic review to assess the outcome measure collected in clinical effectiveness trials of interventions for ankle fracture in adults and the results demonstrate the wide range of methods used as the primary outcome in this type of clinical research study. A similar review has been completed into the PROMs collected in research articles including all types of foot and ankle conditions across specialist journals between 2002 and 2011 [36]. The results of this review differed for the ankle fracture articles, likely because they were collecting all clinical articles in foot and ankle, not just RCTs. They found that the AOFAS was the most widely collected outcome in this decade, followed by the SF-36, and followed by the OMAS. Here, we have not regarded the AOFAS as a PROM as some elements of the outcome measure score rely on clinician assessment [10].

An analysis of publications by decade demonstrates the increase in publication rate of this type of study since the 1980’s. This is in line with results from a study assessing trends in journals dedicated solely to trauma and orthopaedics, which found that the number of these journals rose significantly between 2000 and 2010, indicating an increase in the volume of published works in this clinical field during this time period [37]. The analysis of temporal trends of primary outcome measure use in this type of research article demonstrate the increasing use of the OMAS and radiographic measures as primary outcomes. The increasing trend in the use of an ankle fracture specific outcome measure such as the OMAS is consistent with the increasing trend of patient centred assessment in health care during the same time period [10].

Limitations of this review include the exploratory nature of the research and we are unable to draw conclusions on the effectiveness of management protocols for this patient population. However, the findings will inform further research into outcome assessment in patients with ankle fractures and ultimately improve outcome measurement in clinical research, to enable these important questions to be answered effectively. Furthermore, limited resources meant that a second assessor was only able to review full texts. Due to the large volume of returned search items, a single reviewer completed the initial screening of all titles and abstracts and the data extraction which may have introduced bias. Furthermore, the primary outcome was presumed in cases where this was not explicitly specified may have introduced inconsistency and inaccuracy, which was required in 54 of the 121 records included here. Chan and Bhandari [38] highlighted the variability in reporting RCTs particularly within the field of trauma and orthopaedics. They concluded that researchers within this field often do not report key methodological considerations such as assessor blinding or allocation concealment despite carrying out these actions; this could also be the case in stating the primary outcome measure.

Another limitation is that, for the feasibility of reporting this data, we grouped some assessment methods into single categories, when several methods of measurement were actually used here. For example, the radiographic assessment category included X-Ray, CT scans and other imaging techniques as well as many different assessments and measurements made upon these images, such as quality of reduction, joint congruency, assessments of union and measurements of joint angles and spaces. The variety of measurement techniques used in the outcome measure categories of radiographic assessment, strength, ROM and swelling made exploration of the use of these different measurement methods difficult and is therefore outside of the scope of this review. However, the lack of standardisation in these measurement methods raises concerns regarding generalisability of results across trials that utilise these measures. If these measures continue to be utilised, standardisation of assessment methods is warranted to ensure results are generalisable across studies.

## Conclusions

This review demonstrates the range of outcomes collected in clinical trials of interventions for adults with ankle fracture. Despite overarching current trends towards the use of PROMs in clinical research, this review demonstrates that objective and clinician assessed outcomes remain a commonly analysed primary outcome measure in clinical trials for this patient population. This is despite the lack of consensus on what features or measurements of radiographs are deemed to be important for functional outcome and the value of routine X-Rays in the clinical management of ankle fractures is currently being reviewed [32, 39].

Future research should focus on ascertaining the validity and reliability of these outcome measurements, both the objective clinical measures and the PROMs. Research should examine the value in collecting and analysing clinical measures, such as radiological findings, strength and ROM, over validated patient reported outcome measures. If clinical measures such as these are justified as important outcomes for this patient population, further research is necessary to understand the most valid and reliable measurement methods for purposes of standardisation across studies. Further research is warranted to explore the opinions and views of patients and clinicians in relation to these outcome measures, in order to ensure that research is analysing the factors of most relevance to patients and those which also provide sufficient evidence for clinical decision making. A consensus based study into an appropriate and evidence based core outcome set for interventional trials in ankle fracture management would be advantageous. This would allow for future RCTs in this important research area to be of the highest value during meta-analyses in order to determine the most effective management protocols for this patient population.

## Additional files

Additional file 1: PRISMA checklist referencing page numbers. (PDF 629 kb)

Additional file 2: Search strategy used in review for all databases and registries. (PDF 478 kb)

Additional file 3: Tables showing data extracted from included articles and records during review process. (PDF 939 kb)

## Data Availability

All data analysed during this study are included in this published article and its supplementary information files.

## Abbreviations

AAOS: American Academy of Orthopaedic Surgery
A-FORM: Ankle Fracture Outcome of Rehabilitation Measure
AOFAS: American Orthopaedic Foot and Ankle Surgery
CT: Computed Tomography
DVT: Deep Vein Thrombosis
LEFS: Lower Extremity Functional Scale
MOXFQ: Manchester Oxford Foot Questionnaire
OMAS: Olerud Molander Ankle Score
PROM: Patient Reported Outcome Measure
RCT: Randomised Controlled Trial
ROM: Range of Movement
SF-36: 36-Item Short Form Survey
VAS-Pain: Visual Analogue Scale for Pain
