# Supplementary material for: Primary outcome measures used in interventional trials for ankle fractures: a systematic review
Source: BMC Musculoskelet Disord. 2019 Aug 28;20:388. doi: 10.1186/s12891-019-2770-2 (PMC6712770; doi:10.1186/s12891-019-2770-2)
Supplement: Supplementary file 2 — Search strategy used in review for all databases and registries. (PDF 478 kb) [file 12891_2019_2770_MOESM2_ESM.pdf]

Appendix 2 - Search Strategy

Search initially completed on 02/11/2018

Updated for publication following peer-review on 19/06/2019

Table 1- Search Strategy - Medline

Number	Term	Returns
1	exp ANKLE/	8510
2	exp Ankle Joint/	13849
3	exp Ankle Injuries/	8941
4	exp Fractures, Bone/	167059
5	fracture*.mp.	242117
6	1 or 2 or 3	26960
7	4 or 5	244160
8	6 and 7	5848
9	exp TIBIA/	31611
10	exp Tibial Fractures/	14058
11	exp FIBULA/	8068
12	7 and 11	3434
13	7 and 9	5669
14	8 or 10 or 12 or 13	22739
15	exp Randomized Controlled Trial/	454845
16	exp Controlled Clinical Trial/	542188
17	randomized.mp. or exp RANDOMIZED CONTROLLED TRIALS AS A TOPIC/	672656
18	placebo.mp.	176058
19	randomly.mp.	246332
20	trial.mp.	987780
21	15 or 16 or 17 or 18 or 19 or 20	1303244
22	14 and 21	1146
23	limit 22 to ep=20181101-20190619	12

24	limit 22 to ed=20181101-20190619	47
25	23 or 24	48

Table 2 - Search Strategy - Embase

Number	Term	Returns
1	exp Ankle/	35242
2	exp ankle injury/	13358
3	exp ankle fracture/	6436
4	exp fracture/	310972
5	1 and 4	3172
6	exp distal tibia/	825
7	4 and 6	251
8	exp distal tibia fracture/	899
9	exp distal fibula/	339
10	4 and 9	81
11	exp distal fibula fracture/	160
12	2 or 3 or 5 or 7 or 8 or 10 or 11	16316
13	randomized controlled trial/	555998
14	exp controlled clinical trial/	741317
15	randomized.mp. or exp “randomized controlled trial (topic)” or exp placebo/	1212040
16	randomly.mp.	415131
17	trial.mp. or exp “clinical trial (topic)”/	2038434
18	13 or 14 or 15 or 16 or 17	2539001
19	12 and 18	1328
20	Limit 19 to dd=20181101-20190619	26
21	Limit 19 to dc=20181101-20190619	79
22	20 or 21	80

Table 3 - Search Strategy - CINAHL

Number	Term	Returns
1	(MH “Ankle”)	4476
2	(MH “Ankle Joint”)	5164
3	(MH “Ankle Injuries+”)	5585
4	(MH “Fractures+”)	49656
5	(MH “Tibia”)	6946
6	(MH “Fibula”)	1342
7	S1 OR S2 OR S3	13716
8	S4 AND S7	2351
9	S5 OR S6	7887
10	S4 AND S9	1228
11	S8 OR S10	3310
12	(MH “Tibial Fractures+”)	3190
13	(MH “Fibula Fractures”)	201
14	S11 OR S12 OR S13	5804
15	(MH “Randomized Controlled Trials”) OR (MH “Clinical Trials”)	225490
16	“randomly”	73950
17	“trial”	183772
18	“placebo” OR (MH “Placebos”)	55280
19	S15 OR S16 OR S17 OR S18	379014
20	S14 AND S19	332
21	S14 AND 219 limiters: Published date: 20180101-20191231	42

Table 4 – Search Strategy - AMED

Number	Term	Returns
1	exp Ankle/	1432
2	exp ankle injuries/	1468

3	exp fractures bone/	2539
4	1 and 3	45
5	2 or 4	1513
6	exp Fibula/	316
7	exp Tibia/	1064
8	6 or 7	1277
9	3 and 8	147
10	5 or 9	1613
11	exp Randomized controlled trials/	2210
12	controlled clinical trial.mp.	491
13	exp Clinical trials/ or randomized.mp.	12792
14	randomly.mp.	5994
15	trial.mp.	11004
16	11 or 12 or 13 or 14 or 15	20860
17	10 and 16	146

Table 5 - Search Strategy - Cochrane CENTRAL Trials Register

Number	Term	Returns
#1	MeSH descriptor: [Ankle Joint] explode all trees	663
#2	MeSH descriptor: [Ankle Injuries] explode all trees	665
#3	MeSH descriptor: [Ankle] explode all trees	470
#4	MeSH descriptor: [Tibia] explode all trees	566
#5	MeSH descriptor: [Fibula] explode all trees	70
#6	MeSH descriptor: [Ankle Fractures] explode all trees	121
#7	#1 or #2 or #3 or #4 or #5 or #6	2268
#8	MeSH descriptor: [Fractures, Bone] explode all trees	5398
#9	#8 and #7 with Cochrane publication date from Nov 2018 to Jun 2019, in Trials	13

Table 6 - Search Strategy - ISRCTN Registry

Number	Term	Returns
#1	Ankle Fracture	60

Table 7 - Search Strategy - ClinicalTrials.gov Registry

Number	Term	Returns
#1	Ankle Fracture	117
#2	Limit to "Interventional (Clinical Trial)"	94
