# Supplementary material for: Primary outcome measures used in interventional trials for ankle fractures: a systematic review
Source: BMC Musculoskelet Disord. 2019 Aug 28;20:388. doi: 10.1186/s12891-019-2770-2 (PMC6712770; doi:10.1186/s12891-019-2770-2)
Supplement: Supplementary file 3 — Tables showing data extracted from included articles and records during review process. (PDF 939 kb) [file 12891_2019_2770_MOESM3_ESM.pdf]

Appendix 3 – Data Extraction Table

Reference or Registry number	Interventions Studied	Country	Population & Sample Size (n=)	Was the primary outcome specified?	Primary outcome measure	Secondary outcome measures
Ahl et al. (1986) Early weight bearing of malleolar fractures. *Acta Orthopaedica Scandinavia*	Post-operative Management	Sweden	18 years+ (n=46)	No	ROM	Swelling, OMAS, VAS-function, radiographic assessment
Andersen et al. (2018) Randomized Trial Comparing Suture Button with Single Syndesmotic Screw for Syndesmosis Injury	Operative vs. Operative	Norway	18-70years (n=97)	Yes	AOFAS	OMAS, EQ-5D-5L, VAS-pain, CT scans (radiographic assessment)
Asloum et al. (2014) Internal fixation of the fibula in ankle fractures. A prospective, randomized and comparative study: Plating versus nailing. *Orthopaedics and Traumatology: Surgery & Research*	Operative vs. Operative	France	18-90years (n=71)	Yes	Radiographic assessment	Complications, OMAS, AOFAS
Bauer et al. (1985) Malleolar Fractures: Nonoperative Versus Operative Treatment. A Controlled Study *Clinical Orthopaedics and Related Research*	Operative vs. Non-Operative	Sweden	15-84 years (n=92)	No	Swelling	Muscular atrophy, time to return to work, ROM, radiographic assessment
Bostman et al. (1987) Biodegradeable internal fixation of malleolar fractures. A prospective randomised trial. *The Journal of Bone and Joint Surgery*	Operative vs. Operative	Finland	16-70 years (n=56)	No	Radiographic assessment	Complications, OMAS

Boyle et al. (2014) Removal of the syndesmotic screw after the surgical treatment of a fracture of the ankle in adult patients does not affect one-year outcomes. *The Bone and Joint Journal*	Post-operative Management	New Zealand	16-62years (n=51)	Yes	OMAS	AOFAS, AAOS, VAS-Pain, ROM, radiographic assessment
Braunstein et al. (2016) The value of arthroscopy in the treatment of complex ankle fractures - a protocol of a randomised controlled trial. *BMC Musculoskeletal Disorder*	Operative vs. Operative	Germany	18-65years (n=74 planned population)	Yes	AOFAS	JSSF, OMAS, Karlsson Score, Tegner activity Scale, SF-12, Radiographic assessment, intra-operative assessment, time to return to work/sports, complications
Briet et al. (2015) Weight bearing or non-weight bearing after surgically fixed ankle fractures, the WOW! Study: study protocol for a randomized controlled trial. *Trials*	Post-operative Management	The Netherlands	18-65years (n=225 planned population)	Yes	OMAS	SF-36, ROM, Gait analysis, muscle atrophy, VAS-Pain, return to work/sports, complications
Brink et al. (1996) Stable Lateral Malleolar Fractures Treated with Aircast Ankle Brace and DonJoy R.O.M.-Walker Brace: A prospective Randomized Study. *Foot and Ankle International.*	Conservative Rehabilitation	Denmark	18-84years (n=66)	No	Radiographic assessment	Linear scale of patient satisfaction with intervention (1-5), Linde scoring system, swelling, ROM
Buckley et al. (2018) Single-screw fixation compared with double screw fixation for treatment of medial malleolar fractures: a prospective randomised controlled trial. *J Orthop Trauma.*	Operative vs. operative	Canada	>18 years and <75 years (n=140)	Yes	SF-36	AOFAS, Operating room time.
Bucholz et al. (1994) Fixation with Bioabsorbable Screws for the Treatments of Fractures of the Ankle. *The Journal of Bone and Joint Surgery*	Operative vs. Operative	USA	21-59years (n=155)	No	Radiographic assessment	OMAS, complications

Buttenschoen et al. (2001) The influence of vacuum-assisted closure on inflammatory tissue reactions in the postoperative course of ankle fractures. *Foot and Ankle Surgery*	Operative vs. Operative	Germany	27-74years (n=35)	No	Blood biochemistry analysis	Further blood biochemistry analysis methods
Caschman et al. (2004) Efficacy of the A-V Impulse System in the Treatment of Posttraumtic Swelling Following ankle Fracture. *J Orthop Trauma*	Pre-operative management	UK&Ireland	18years+ (n=64)	Yes	Swelling assessment	Time taken for swelling to resolve ready for surgery, complications, length of stay, VAS-Pain, Analgesia usage
Christie and Wiilloughby (1990) The effect of interferential therapy on swelling following open reduction and internal fixation of ankle fractures. *Physiotherapy Therapy and Practice*	Post-operative management	Australia	15-61years (n=24)	Yes	Swelling assessment	None.
Dehghan et al. (2016) Early Weightbearing and Range of Motion Versus Non-Weightbearing and Immobilization After Open Reduction and Internal Fixation of Unstable ankle Fracture: A Randomized Controlled Trial. *J Orthop Trauma*	Post-operative management	Canada	18years+ (n=110)	Yes	Time to return to work	ROM, SF-36, OMAS, complications
Dijkema et al. (1993) Surgical treatment of fracture-dislocations of the ankle joint with biodegradeable implants: a prospective randomized study. *The Journal of Trauma*	Operative vs. Operative	The Netherlands	16-70years (n=43)	No	OMAS	Semi-quantitative opinion of surgeon and patient on ankle function, radiographic assessment
Dingemans et al. (2018) Routine versus on demand removal of the syndesmotic screw; a protocol for an international randomised controlled trial (RODEO-trial) *BMC Musculoskeletal Disorders*	Post-operative management	The Netherlands	"adult" (n=194 planned population)	Yes	OMAS	AOFAS, Vas-Pain, ROM, Post-op infections, Radiographic assessment of syndesmosis, EQ-5D-5L and Resource Use/HE Analysis

Dogra and Rangan (1999) Early mobilisation versus immobilisation of surgically treated ankle fractures. Prospective randomised control trial. *Injury, Int J. Care Injured*	Post-operative management	UK&Ireland	16-65years (n=52)	No	ROM	VAS-Pain, Gait analysis, OMAS
Egol et al. (2000) Functional outcome of surgery for fractures of the ankle. *The Journal of Bone and Joint Surgery (Br)*	Post-operative management	USA	15-77years (n=60)	No	Return to work	SF-36, radiographic assessment
Erdem, et al. (2014) Comparison of Lag Screw Versus Buttress Plate Fixation of Posterior Malleolar Fractures. *Foot and Ankle International*	Operative vs. Operative	Turkey	21-51years (n=40)	No	ROM	Radiographic assessment
Ewald et al. (2015) Does Ankle Aspiration for Acute Ankle Fracture Results in Pain Relief? A Prospective Randomized Double-Blinded Placebo Controlled Trial. *J Orthop Trauma*	Pre-operative Management	USA	Not specified range, mean 52years (n=124)	Yes	Analgesia usage	None
Finsen et al. (1989) Early Postoperative Weight-Bearing and Muscle Activity in Patients Who Have a Fracture of the Ankle. *The Journal of Bone and Joint Surgery*	Post-operative Management	Norway	Not specified range, mean 41years (n=56)	No	Radiographic assessment	Return to work
Franke et al. (2008) The dynamic vacuum orthosis: a functional and economical benefit? *International Orthopaedics*	Post-operative Management	Germany	18-65years (n=27)	Yes	ROM	VAS-patient satisfaction, SF-12, time to return to work, economic analysis on a sample of patients
Ge et al. (2017) Preliminary effect of posterolateral ankle arthroscopy for ankle fractures in elderly population. *Int J Clin Exp Med*	Operative vs. Operative	China	>60years (n=68)	No	Operation time	Intra-op bleeding and complications, time in recovery, McGuire scoring system, VAS-Pain

Georgiannos et al. (2017) Fragility fractures of ankle in the elderly: Open reduction and internal fixation versus tibio-talo-calcaneal nailing: Short-term results of a prospective randomized-controlled trial. *Injury*	Operative vs. Operative	Greece	>70years (n=87)	Yes	Intra and post-operative complications	Length of stay, mobility status, reoperation rate, OMAS, radiographic assessment
Ginandes and Rosenthal (1999) Using hypnosis to accelerate the healing of bone fractures: a randomised controlled pilot study. *Alternative Therapies*	Conservative Rehabilitation	USA	18years+ (n=12)	No	Radiographic assessment	Surgeon assessment of fracture healing, hypnotic induction scale
Gorodetskyi et al. (2010) Use of Noninvasive Interactive Neurostimulation to Improve Short-Term Recovery in Patients with Surgically Repaired Bimalleolar Ankle Fractures: A Prospective, Randomised Clinical Trial. *The Journal of Foot & Ankle Surgery*	Post-operative Management	Russia	20-60years (n=60)	No	VAS-Pain	ROM, swelling assessment
Handolin et al. (2005) No long-term effects of ultrasound therapy on bioabsorbable screw-fixed lateral malleolar fracture. *Scandinavian Journal of Surgery*	Post-operative management	Finland	22-66years (n=16)	No	Radiographic assessment	OMAS, clinician assessment of ankle joint
Handolin et al. (2005) Effect of ultrasound therapy on bone healing of lateral malleolar fractures of the ankle joint fixed with bioabsorbable screws. *J Orthop Sci*	Post-operative Management	Finland	18-65years (n=22)	No	Radiographic assessment	None
Hedström et al. (1994) Early Postoperative Ankle Exercise. A Study of Postoperative Lateral Malleolar Fractures. *Clinical Orthopaedics and Related Research*	Post-operative management	Sweden	15-71years (n=53)	No	ROM	Linear score 0-100, OMAS, radiographic assessment

Hoelsbrekken et al. (2013) Nonoperative Treatment of the Medial Malleolus in Bimalleolar and Trimalleolar Ankle Fractures: A Randomized Controlled Trial. *J Orthop Trauma*	Operative vs. operative	Norway	18years+ (n=100)	Yes	OMAS	AOFAS, VAS-pain, Radiographic assessment
Hoiness and Stromsoe. (2004) Tricortical Versus Quadricortical Syndesmosis Fixation in Ankle Fractures. *J Orthop Trauma*	Operative vs. operative	Norway	Not specified range, mean 42years (n=64)	No	OMAS	ROM, Radiographic assessment
Honigmann et al. (2007) Aftertreatment of malleolar fractures following ORIF - functional compared to protected functional in a vacuum-stabilized orthesis: a randomized controlled trial. *Arch Orthop Trauma Surg*	Post-operative management	Switzerland	16-65years (n=45)	Yes	OMAS	SF-12, return to work, VAS for satisfaction with treatment and walking security, ROM, swelling, muscle atrophy, complications, length of stay
Jansen et al. (2018) Active controlled motion in early rehabilitation improves outcome after ankle fractures: a randomized controlled trial. *Clinical Rehabilitation*	Conservative Rehabilitation	Germany	22-73years (n=50)	No	ROM	VAS-function of foot and ankle, Philip Score, Mazur Score, AOFAS, Gait analysis,
Joukainen et al. (2007) Bioabsorbable screw fixation of ankle fractures. *J Orthop Sci*	Operative vs. Operative	Finland	14-73years (n=62)	No	Radiographic assessment	Clinical Assessment, OMAS, ROM, complications
Kankare et al. (1995) Malleolar fractures in alcoholics treated with biodegradable internal fixation: 6/16 reoperations in a randomized study. *Acta Orthopaedica Scandinavia*	Operative vs. Operative	Finland	29-62years (n=29)	No	OMAS	Radiographic assessment, complications

Kankare et al. (1996) Biodegradable self-reinforced polyglycolide screws and rods in the fixation of displaced malleolar fractures in the elderly. *Annales Chirurgiae et Gynaecologiae*	Operative vs. Operative	Finland	65-90years (n=37)	No	OMAS	Radiographic assessment, complications
Kearney et al. (2019) Cast versus brace in the rehabilitation of patients treated for an ankle fracture: protocol for the UK study of ankle injury rehabilitation (AIR) multicentre randomised controlled trial. *BMJ Open.*	Rehabilitation of operatively and non-operatively managed patients (mixed)	UK&Ireland	18 years + (n=478)	Yes	OMAS	M-OXFQ, EQ-5D-5L, Disability Rating Index, complications and cost-utility.
Keene et al. (2016) The Immediate Effects of Different Types of Ankle Support Introduced 6 Weeks After Surgical Internal Fixation for Ankle Fracture on Gait and Pain: A Randomized Crossover Trial. J*ournal of Orthopaedic & Sports Physical Therapy*	Post-operative management	UK&Ireland	19-77years (n=18)	Yes	Gait analysis	None
Kimmel et al. (2012) Rest easy? Is bed rest really necessary after surgical repair of an ankle fracture? *Injury*	Post-operative management	Australia	18-80years (n=104)	Yes	Length of stay	Destination of discharge, analgesia requirements, wound complications
Konrad et al. (2005) Tourniquets May Increase Post Operative Swelling and Pain after Internal Fixation of Ankle Fractures. *Clinical Orthopaedics and Related Research*	Operative vs Operative	Germany	18-70years (n=54)	No	Swelling assessment	ROM ,VAS-pain, length of stay, wound complications/healing issues
Kortekangas et al. (2014) Syndesmotic Fixation in Supination-External Rotation Ankle Fractures: A Prospective Randomized Study. *Foot and Ankle International*	Operative vs. Operative	Finland	Not specified range, mean 43years (n=24)	No	OMAS	VAS-pain, SF-36, further procedures, ROM, radiographic analysis

Kortekangas et al. (2015) A prospective randomised study comparing TightRope and syndesmotic screw fixation for accuracy and maintenance of syndesmotic reduction assessed with bilateral computed tomography. *Injury*	Operative vs. Operative	Finland	20-79years (n=43)	Yes	Radiographic analysis	OMAS, VAS-Pain and function, AF-36, Foot and ankle outcome score, further procedures, ROM
Kortekangas et al. (2019) Three week versus six-week immobilisation for stable Weber B Type ankle fractures: randomised, multicentre, non-inferiority clinical trial. *British Medical Journal.*	Conservative rehabilitation	Finland	16+years (n=247)	Yes	OMAS	FAOS, VAS function and pain, RAND-35 Item health questionnaire, ROM, malunion and fracture union measured radiographically.
van Laarhoven et al. (1996) Postoperative treatment of internally fixed ankle fractures. A prospective randomised study. *The Journal of Bone and Joint Surgery (Br).*	Post-operative Management	The Netherlands	15-77years (n=55)	No	Modified OMAS	VAS-function, clinician assessment, ROM, radiographic assessment, presence of OA
Laflamme et al. (2015) A Prospective Randomised Multicenter Trial Comparing Clinical Outcomes of Patients Treated Surgically With a Static or Dynamic Implant for Acute Ankle Syndesmosis Rupture. *Journal of Orthopaedic Trauma*	Operative vs. Operative	Canada	18-65years (n=70)	Yes	OMAS	AOFAS, VAS-pain, ROM, swelling, time to return to work, radiographic assessment
Lamontagne et al. (2002) Surgical Treatment of a Displaced Lateral Malleolus Fracture: The Antiglide Technique Versus Lateral Plate Fixation. *Journal of Orthopaedic Trauma*	Operative vs. Operative	Canada	18-69years (n=193)	No	OMAS	Complications

Lehtonen et al. (2003) Use of a Cast Compared with a Functional Brace after Operative Treatment of an Ankle Fracture. A Prospective Randomised Study. *The Journal of Bone and Joint Surgery*	Post-operative management	Finland	Not specified, mean 41years (n=100)	No	AOFAS	Intraoperative blood loss, duration of operation, time to initiate restricted weight bearing ambula+F45tion, length of hospital stay and rates of complications. Ankle swelling, calf atrophy, ROM, OMAS, Kaikkonen score, time to return to work
Li et al. (2018) Percutaneous compression cannulated screw fixation for ankle fractures. *Int J Clin Exp Med.*	Operative vs. Operative	China	Range not specified, mean 35.2 (n=100)	Yes	Fracture healing assessed radiographicall y	AOFAS, intraoperative blood loss, duration of operation, time to initiate restricted weight bearing ambulation, length of hospital stay and rates of complications.
Li et al. (2016) Minimally invasive treatment of medial malleolus fracture by implanting a hollow screw under C-arm X-ray. *Int J Clin Exp Med*	Operative vs. Operative	China	19-61years (n=23)	No	Length of stay	Radiographic assessment, ankle function score described by criteria of Majeed (unable to assess the components of this outcome measure from information given)

Lin et al. (2008) Manual therapy in addition to physiotherapy does not improve clinical or economic outcomes after ankle fracture. *J Rehabil Med*	Conservative Rehabilitation	Australia	Not specified range, mean 41years (n=94)	Yes	LEFS	Assessment of Quality of Life, Gait analysis, ROM, VAS-Pain on weight bearing, measures of participation and return to work/sports, satisfaction with rehabilitation treatment using VAS scale, Global perceived treatment effect on 11-point scale, number of days to pain free walking, complications, health economic analysis
Maffulli et al. (1993) Use of a Tourniquet in the Internal Fixation of Fractures of the Distal Part of the Fibula. A Prospective Randomized Trial. *The Journal of Bone and Joint Surgery Incorporated*	Operative vs. Operative	UK&Ireland	18-60years (n=80)	No	Wound complications	Radiographic assessment, clinician assessment of healing status,
Makwana et al. (2001) Conservative versus operative treatment for displaced ankle fractures in patients over 55 years of age. A prospective randomised study. *The Journal of Bone and Joint Surgery (Br)*	Operative vs. non-operative	UK&Ireland	>55years (n=47)	No	OMAS	VAS-Pain, swelling measurements, ROM, instability assessment performed by clinician, wound complications, scar tenderness and prominence of metal work, walking distance, subjective patient satisfaction, radiographic assessment

Matthews et al. (2018) Early Motion and directed exercise (EMADE) versus usual care post ankle fracture fixation: study protocol for a pragmatic randomised controlled trial. *Trials*	Post-operative management	UK&Ireland	18years+ (n=156)	Yes	OMAS	A-FORM, EQ-5D, Physical Activity Record Scale (PARS), Clinical Physical Activity Questionnaire (CPAQ), use of walking aid, returns to work and return to driving, X-Ray findings and adverse events/complications
Mayich et al. (2013) Role of Patient Information Handouts Following Operative Treatment of Ankle Fractures: A Prospective Randomized Study. *Foot and Ankle international.*	Post-operative management	Canada	18-65years (n=40)	Yes	Likert scale of satisfaction with intervention	radiographic assessment, OMAS
Mittal et al. (2017) Surgery for Type B Ankle Fracture Treatment: a combined Randomised and Observational Study (CROSSBAT). BMJ Open	Operative vs. non-operative	Australia	18-65years (n=160)	Yes	AAOS	SF-12, complications, number of physiotherapy sessions
Mora et al. (2002) The Role of Pulsatile Cold Compression in Edema Resolution Following Ankle Fractures: A Randomized Clinical Trial. *Foot and Ankle International.*	Pre-operative management	USA	Not specified, mean 33years (n=24)	No	Swelling measurements	Score of satisfaction (1-4),
Moseley et al. (2015) Rehabilitation After Immobilization for Ankle Fracture. The EXACT Randomized Clinical Trial. *Journal of American Medical Association*	Rehabilitation of operatively and non-operatively managed patients (mixed)	Australia	Not specified range, mean 45years (n=214)	Yes	LEFS	Assessment of QoL, Number of days to pain free walking, time to return to work, percentage return to pre-injury activity, ROM, VAS-pain, gait analysis, activity level, global perceived effect of treatment, economic analysis, complications

Moseley et al. (2005) Passive Stretching Does Not Enhance Outcomes in Patients With Plantarflexion Contracture After Cast Immobilization for Ankle Fracture: A Radomized Controlled Trial. *Arch Phys Med Rehabil*	Conservative Rehabilitation	Australia	Not specified range, mean 45years (n=150)	Yes	LEFS	Measures of ankle stiffness, ROM, VAS-Pain, Return to work and normal activities, gait analysis, global perceived effects of treatment on linear scale, satisfaction with intervention, number of physiotherapy sessions
Nilsson et al. (2009) Effects of a training program after surgically treated ankle fracture: a prospective randomised controlled trial. *BMC Musculoskeletal Disorders.*	Post-operative management	Sweden	Not specified range, mean 33years (n=110)	Yes	OMAS	SF-36, gait analysis, ROM, muscle strength tests, radiographic assessments,
Noh et al. (2012) Outcomes of Operative Treatment of Unstable Ankle Fractures: A Comparison of Metallic and Biodegradable Implants. *The Journal of Bone and Joint Surgery Incorporated*	Operative vs. Operative	Korea	>16years (n=102)	No	AOFAS	SMFA, radiographic assessment
Pakarinen et al. (2011) Syndesmotic Fixation in Supination-External Rotation Ankle Fractures: A Prospective Randomized Study. *Foot and Ankle International.*	Operative vs. Operative	Finland	25-67years (n=140)	No	OMAS	SF-36, radiographic assessment, ROM
Pan et al. (2018) Surgical effects and prognosis of non-rigid internal fixation for ankle fractures combined with tibiofibular syndesmotic injuries. *In J Clin Exp Med*	Operative vs. Operative	China	Range not specified, means 36.4 and 37.1 in each group (n=90)	No	Duration of operation	Operation angle of fixation for distal tibiofibular joint, time to achieve complete weight-bearing ambulation, post-

						operative complication rate, AOFAS.
Park et al. (2018) Comparison of Clamp Reduction and Manual Reduction of Syndesmosis in Rotational Ankle Fractures: A Prospective Randomized Trial. *The Journal of Foot and Ankle Surgery.*	Operative vs. Operative	Korea	19-83years (n=85)	No	ROM	VAS-Pain, OMAS, Radiographic assessments
Phillips et al. (1985) A Prospective, Randomized Study of the Management of Severe Ankle Fractures. *The Journal of Bone and Joint Surgery Incorporated.*	Operative vs. Non-operative	USA	15-78years (n=142)	No	Self-devised scoring system (compared to that of Mazur) – referenced here as Phillip score.	Radiographic assessment
Pritchett (1993) Rush Rods Versus Plate Osteosyntheses for Unstable ankle Fractures in the Elderly. *Orthopaedic Review*	Operative vs. Operative	USA	65-84years (n=50)	No	Ankle strength tests	ROM, Radiographic assessments
Rashid et al. (2013) To evaluate the efficiency of Mobilization Techniques in Post-Traumatic stiff ankle with and without Paraffin Wax Bath. *Pak J Med Sci*	Conservative Rehabilitation	Pakistan	20-60years (n=37)	No	ROM	VAS-Pain
Rowley et al. (1986) A prospective trial comparing operative and manipulative treatment of ankle fractures. *The Journal of Bone and Joint Surgery.*	Operative vs. Non-operative	UK&Ireland	16-70 (n=42)	No	ROM	Gait analysis, radiographic assessment

Sanders et al. (2012) Operative Versus Nonoperative Treatment of Unstable Lateral Malleolar Fractures: A Randomized Multicenter Trial. *J Orthop Trauma*	Operative vs. non-operative	Canada	Skeletally mature up to 65years (n=81)	Yes	SF-36 – physical component score	OMAS – physical component score, further procedures, complications
Shannon et al. (2017) Allgower-Donati Versus Vertical Mattress Suture Technique Impact on Perfusion in Ankle Fracture Surgery: A Randomized Clinical Trial Using Intraoperative Angiography. *J Orthop Trauma*	Operative vs. Operative	USA	20-91years (n=30)	Yes	Mean perfusion rates measured radiographicall y	Other radiographically measured perfusion impairment tests
Shi et al. (2018) Comparison of the direct and indirect reduction techniques during the surgical management of posterior malleolar fractures.	Operative vs. Operative	China	Range not specified, mean 49.0 and 48.1 in each group (n=116)	No	Fracture reduction via CT Scan Radiographical ly	AOFAS, ROM, VAS-pain.
Siddique et al. (2005) Early Active Mobilization Versus Cast Immobilization in Operatively Treated ankle Fractures. A Prospective Analysis of Early Functional Recovery. *European Journal of Trauma*	Post-operative management	UK&Ireland	16-60years (n=44)	No	ROM	OMAS, Radiographic assessment
Sim et al. (2019) Effect of Hospital Length of Stay on Tourniquet Use during Internal Fixation of Ankle Fractures: Randomized Controlled Trial. *The Journal of Foot and Ankle Surgery.*	Operative vs. operative	UK&Ireland	18+ years (n=188)	Yes	Length of Stay	Duration of operation, Occurrence of intra-operative and post-operative complications.
Sondenaa et al. (1986) Immobilization of operated ankle fractures. *Acta Ortho Scand*	Post-operative management	Norway	16-59years (n=53)	No	Strength	ROM, swelling, pain
Springer et al. (1998) Resorbable rods and screws for the fixation of ankle fractures. A prospective randomized clinical trial. *Unfallchirurg*	Operative vs. Operative	The Netherlands	16-75years (n=50)	No	Radiographic assessments	OMAS, complications

Strömsöe et al. (1995) The repair of a ruptured deltoid ligament is not necessary in ankle fractures. *Journal of Bone and Joint Surgery (Br)*	Operative vs. Operative	Norway	17-75years (n=50)	No	Radiographic assessments	Length of stay, complications, further procedures
Stuart et al. (1989) Comparative study of functional bracing and plaster cast treatment of stable lateral malleolar fractures. *Injury*	Conservative rehabilitation	UK&Ireland	Not specified "adults" (n=40)	No	Radiographic assessments	Ankle comfort using VAS scale, swelling, ROM, symptomatology (not stated how measured)
Sultan et al. (2014) Compression stockings in the management of fractures of the ankle. A randomised controlled trial. *The Bone and Joint Journal.*	Rehabilitation of operatively and non-operatively managed patients (mixed)	UK&Ireland	16-79years (n=90)	Yes	OMAS	SF-12, AOFAS, DVT incidence, swelling, ROM
Sun et al. (2014) A Prospective, randomised trial comparing the use of absorbable and metallic screws in the fixation of distal tibiofibular syndesmosis injuries. *The Bone and Joint Journal.*	Operative vs. Non-Operative	China	18-72years (n=168)	No	Baird Score	ROM, Pain, radiographic assessment, complications
Takao et al. (2004) Diagnosis and Treatment of Combined Intra-articular Disorders in Acute Distal Fibular Fractures. *The Journal of Trauma Injury, Infection, and Critical Care*	Operative vs. Operative	Japan	20-64years (n=72)	No	AOFAS	Radiographic assessment, clinical assessment
Thordarson et al. (2001) The role of ankle Arthroscopy on the Surgical Management of Ankle Fractures. *Foot and Ankle International.*	Operative vs. Operative	USA	Not specified range, mean 29years (n=19)	No	Intra-operative assessment	AAOS

Thordarson et al. (1997) International Pneumatic Pedal Compression and Edema Resolution After Acute Ankle Fracture: A Prospective, Randomized Study. *Foot and Ankle International.*	Post-operative rehabilitation	USA	Not specified adult population (n=30)	Yes	Swelling	None
Thordarson et al. (2001) Bioabsorbable Versus Stainless Steel Screw Fixation of the Syndesmosis in Pronation-Lateral Rotation Ankle Fractures: A Prospective Randomized Trial. *Foot and Ankle International.*	Operative vs. Operative	USA	Not specified range, means of each group 32 and 24years (n=32)	No	Clinical exam – non-descript	Questionnaire (no reference or description given), radiographic assessment
Tropp et al. (1995) Ankle Performance after Ankle Fracture: A Randomized Study of Early Mobilization. *Foot and Ankle International.*	Post-operative management	Sweden	19-60years (n=30)	No	OMAS (no description of changes made in paper)	Measurement of atrophy, swelling, ROM, strength, wound healing via clinical assessment, complications, radiographic assessment
Tsukada et al. (2013) Locking versus non-locking neutralization plates for treatment of lateral malleolar fractures: a randomized controlled trial. *International Orthopaedics.*	Operative vs. Operative	Japan	Not specified range, mean 40years (n=52)	Yes	Radiographic assessment of union	SF-36, clinical confirmation of bone union, complications
Tuckett et al. (2018) Transarticular tibiotalocalcaneal nailing versus open reduction and internal fixation for treatment of the elderly ankle fracture: protocol for a multicentre randomised controlled trial. *BMJ Open.*	Operative vs. operative	Australia	50 years + (n=110)	Yes	Complication rate	Length of Stay, Non-union, blood transfusion, medical complications, mobility at discharge, discharge destination, AOFAS, OMAS, Secondary interventions

						required, mortality rate and EQ-5D-5L
van den Berg et al. (2018) Functional bracing treatment for stable type B ankle fractures. *Injury*	Conservative Rehabilitation	The Netherlands	18-74 (n=44)	Yes	OMAS	VAS comfort and pain, AAOS, EQ-5D and ROM
van der Velde et al. (2013) Reducing the potential for tourniquet-associated reperfusion injury. *European Journal of Emergency Medicine*	Operative vs. Operative	UK&Ireland	Not specified adult (n=16)	Yes	pH changes via blood gas/biochemis try analysis	Serum potassium sodium, lactate and calcium concentration, PaO2, PaCO2, base excess, HCO3, SaO2
Verhage et al. (2017) Medium-sized posterior fragments in AO Weber-B fractures, does open reduction and fixation improve outcome? The POSTFIX-trial protocol, a multicenter randomized clinical trial. *BMC Musculoskeletal Disorders.*	Operative vs. operative	The Netherlands	18-70years (n=84)	Yes	AAOS	AOFAS, OMAS< Radiographic assessment, VAS-Pain, ROM, EQ-5D-5L
Vidovic et al. (2017) Posterior fragment in ankle fractures: anteroposterior vs posteroanterior fixation. *Injury*	Operative vs. operative	Croatia	33-71 years (n=48)	No	Quality of reduction radiographicall y	Rom and complication rates.
Vioreanu et al. (2007) Early Mobilization in a Removable Cast Compared with Immobilization in a Cast After Operative Treatment of Ankle Fractures: A Prospective Randomized Study. *Foot and Ankle International*	Post-operative management	UK&Ireland	14-65years (n=66)	No	Ankle swelling	Muscle atrophy, ROM, OMAS, Radiographic assessment, AOFAS, time to return to work, SF-36

White et al. (2016) A prospective randomised controlled trial of the fibular nail versus standard open reduction and internal fixation for fixation of ankle fractures in elderly patients. *The Bone and Joint Journal*	Operative vs. Operative	UK&Ireland	65-93years (n=100)	Yes	OMAS	Complications, SFMA, VAS for satisfaction with scar, economic evaluation
Willett et al. (2016) Close Contact Casting vs Surgery for Initial Treatment of Unstable Ankle Fractures in Older Adults. A Randomized Clinical Trial. *Journal of American Medical Association*	Operative vs. non-operative	UK&Ireland	>60years (n=620)	Yes	OMAS	SF-12, EQ-5D-3L, Pain, Patient satisfaction with treatment, economic evaluation, time spent non-weight bearing, ROM, Timed get up and Go test, complications, radiographic assessment
Winge et al. (2018) Wound complications after ankle surgery. Does compression treatment work? A randomized, controlled trial. *European Journal of Trauma and Emergency Surgery.*	Post-operative management	Denmark	18+ years (n=153)	Yes	Infection	Necrosis and Wound dehiscence
Xian et al. (2018) Novel Elastic Syndesmosis Hook Plate Fixation Versus Routine Screw Fixation for Syndesmosis Injury. *The Journal of Foot and Ankle Surgery*	Operative vs. Operative	China	18-65years (n=68)	No	Radiographic assessment	Wound complications, VAS-Pain, ROM, time to return to work/usual activities
Zhan et al. (2016) Anterior-inferior tibiofibular ligament anatomical repair and augmentation versus trans-syndesmosis screw fixation for the syndesmotic instability in external-rotation type ankle fracture with posterior malleolus involvement: A prospective and comparative study. *Injury*	Operative vs. operative	China	19-67 (n=68)	No	Radiographic assessment, quality of reduction	Radiographic assessment (syndesmosis diastasis recurrence), VAS-pain, return to work, OMAS, ROM, wound complications

Kearney (2015) AIR: Ankle injury rehabilitation. *ISRCTN17809322* https://www.isrctn.com/ISRCTN17809322	Rehabilitation of operatively and non-operatively managed patients (mixed)	UK&Ireland	16years+ (n=50 planned)	Yes	MOXFQ	complications, EQ-5D-5L, OMAS, healing via radiographic assessment
Tbaily (2015) Does early mobilisation after ankle fracture surgery enhance recovery? *ISRCTN15497399* https://www.isrctn.com/ISRCTN15497399	Post-operative management	UK&Ireland	16years+ (n=246)	Yes	OMAS	ROM, weight-bearing status, EQ-5D-5L, healing status, complications, return to usual activites
Keene (2018) Ankle fracture Treatment: Enhancing Rehabilitation - The AFTER Study. *ISRCTN16612336* http://www.isrctn.com/ISRCTN16612336	Rehabilitation of operatively and non-operatively managed patients (mixed)	UK&Ireland	"Adult" (n=48 planned sample)	Yes	Feasibility of recruitment, retention and follow up	OMAS, LEFS, VAS-Pain, EQ-5D-5L, Fear of Falls using Falls Efficacy Scale International (short), Self Efficacy Exercises Score, Return to usual activities, Walking aid use and distance, Medication use, Resource Use, out of pocket expenses, Adverse events, adherence to exercises, ROM, strength, short physical performance battery plus a qualitative study on a small sample of patients.

Hennessy (2012) Does the Geko nerve stimulator reduce Deep Vein Thrombosis (DVT) and improve healing in ankle fractures? *ISRCTN95441725* https://www.isrctn.com/ISRCTN95441725	Post-operative management	UK&Ireland	18years+ (n=246)	Yes	DVT Rate	Time to union radiographically assessed, MOXFQ and time to return to work
Hing (2010) A trial comparing weight bearing to non-weight bearing following ankle fracture fixation. *ISRCTN33416471* https://www.isrctn.com/ISRCTN33416471	Post-operative management	UK&Ireland	18-70years (n=76)	Yes	OMAS	EQ-5D-5L, ROM,PROMS (not specified), complications, time lost from work, length of stay and physiotherapy intervention required, radiographic assessment of reduction and fracture union, bony tenderness on palpation, pain free weight bearing
Kenyon (2008) Outcomes using biodegradeable fixation materials for fractures of the ankle. *ISRCTN67973353* https://www.isrctn.com/ISRCTN67973353	Operative vs. Operative	UK&Ireland	18-65years (n=146)	Yes	AOFAS	VAS-pain, OMAS, time of injury until operation, tourniquet time, poast medical history, Co-morbidities, smoking history, time non-weight bearing following surgery, further surgery, complications
Encore Medical (2016) CMF Bone Stimulation as Adjunct to Surgical Treatment of Ankle Fractures *NCT02688855* https://clinicaltrials.gov/ct2/show/NCT02688855	Post-operative management	USA	21years + (n=217)	Yes	OMAS	Composite safety endpoint including radiographic assessment, further procedures or revisions, complications

Tejwan (2016) Early Weight Bearing for Unstable Ankle Fractures Undergoing Operative Stabilization. *NCT02779244* https://clinicaltrials.gov/ct2/show/NCT02779244	Post-operative management	USA	21years+ (n=132)	Yes	OMAS	None
Anderson (2017) Arthroscopic Assisted Open Reduction Internal Fixation Versus Open Reduction Internal Fixation in Ankle Fractures. *NCT03084263* https://clinicaltrials.gov/ct2/show/NCT03084263	Operative vs. Operative	USA	18-65years (n=0 – no participants enrolled as study withdrawn)	Yes	AOFAS	FAAM, return to sports, complications, Pain scores
Rijnstate Hospital (2016) Post Operative Quality of Life and Pain in ankle Fractures: Cast Versus Functional Treatment. NCT02823275 https://clinicaltrials.gov/ct2/show/NCT02823275	Post-operative management	The Netherlands	18-65years (n=13)	Yes	VAS-Pain	Analgesia usage
Sanders (2014) Open Reduction Syndesmosis Tightrope Versus Screw Fixation. *NCT02199249* https://clinicaltrials.gov/ct2/show/NCT02199249	Operative vs. Operative	Canada	18 years + (n=103)	Yes	CT and Radiographic outcome (reduction)	VAS-pain, functional performance using EQ-5D, Foot and Ankle Disability Index (FADI) and AAOS Hindfoot Score, return to work.
Benedikte Wendt Ræder (2016) Ziptight or Tricortical Screw Fixation of Acute Tiobiofibular Syndesmotic Injury (S16) *NCT02930486* https://clinicaltrials.gov/ct2/show/NCT02930486	Operative vs. Operative	Norway	18-70 years (n=120)	Yes	Modified AOFAS (OTA Score)	MOXFQ, ROM Dorsiflexion, HRQoL using EQ-5D, Radiographic assessment (syndesmotic distance), VAS-pain
Carter (2017) Medial Malleolus: operative or Non-operative (MOON) *NCT03362229* https://clinicaltrials.gov/ct2/show/NCT03362229	Operative vs. Operative	UK&Ireland	16years + (n=154)	Yes	OMAS	ED-5D, MOXFQ, tourniquet/operation time, VAS-pain

Pakarinen (2012) Unimalleolar Versus Bimalleolar Fixation in Bi- or Trimalleolar Ankle Fracture. *NCT01757951* https://clinicaltrials.gov/ct2/show/NCT01757951	Operative vs. Operative	Finland	16years + (n=126)	Yes	OMAS	AAOS FAOS, VAS-Pain, SF36, Joint congruence and healing/union measured radiographically, complications.
Koval (2015) Fibular Fixation in Ankle Fractures: Plate verses Nail. *NCT02507193* https://clinicaltrials.gov/ct2/show/NCT02507193	Operative vs. Operative	USA	18years + (n=60)	Yes	OMAS	None
Giannoudis (2009) Comparison of Biodegradable & Metal Plates for Fixing Ankle Fracture. *NCT00864877* https://clinicaltrials.gov/ct2/show/NCT00864877	Operative vs. Operative	UK&Ireland	18-60years (n=0 – not completed and no sample size given)	Yes	OMAS	None
Tornetta 3rd (2008) A Multicenter Randomized Controlled Trial Comparing Antiglide and Lateral Plate Fixation in Ankle Fractures. *NCT00718302* https://clinicaltrials.gov/ct2/show/NCT00718302	Operative vs. Operative	USA	18-85years (n=249)	Yes	Non-palpable hardware (clinician assessment)	Normal peroneal tendons (assessed by clinician), AOFAS, SMFA Score and SMFA Bother Index
Schønnemann (2015) Preoperative Treatment of Malleolar Fractures. *NCT02444468* https://clinicaltrials.gov/ct2/show/NCT02444468	Pre-operative management	Finland	18years + (n=96)	Yes	Time to surgery	None
Kiner (2014) Surgical Versus Nonsurgical Treatment of Fibular Fractures: A Prospective Randomized Study. *NCT02032966.* https://clinicaltrials.gov/ct2/show/NCT02032966	Operative vs. Operative	USA	18years + (n=150)	Yes	Radiographic assessment (time to union)	Time to weight-bearing, complications and reoperation rate, SF-36, Foot Function Index (FFI)
Brink (2006) Inion OTPS Biodegradebale Fixation System for the Ankle. *NCT00300989* https://clinicaltrials.gov/ct2/show/NCT00300989	Operative vs. Operative	Finland	18-60years (n=43)	Yes	Kaikkonen Functional Score	OMAS, Radiographic assessment, Return to activities, SF-36, VAS-

						pain, wound healing via clinician assessment
Amanatullah (2017) Clinical Trial Evaluating Acutrak Headless Compression Screw Fixation of Medial Malleolus Fractures *NCT03061279* https://clinicaltrials.gov/ct2/show/NCT03061279	Operative vs. Operative	USA	18 years + (n=500)	Yes	Removal rate and further procedures	Fracture non-union via radiographic assessment, PROMIS Score, Hardware related pain
Kim (2014) Early Weightbearing Versus Non-weightbearing After Operative Treatment of an Ankle Fracture. *NCT02029170* https://clinicaltrials.gov/ct2/show/NCT02029170	Post-operative management	Korea	18-65 years (n=192)	Yes	OMAS	Time to return to activity and full weight bearing, VAS-satisfaction, hardware failure, reduction loss or non-union assessed radiographically.
Leong (2014) Outcome of Rehabilitation Following Internally Fixed Ankle Fractures *NCT02160197* https://clinicaltrials.gov/ct2/show/NCT02160197	Post-operative management	UK&Ireland	18-50 years (n=105)	Yes	MOXFQ	None
Krause (2018) Comparing Function, Pain and Return to Work in Conservative Versus Surgical Treated Stable Lateral Malleolar Fractures. *NCT03587571* https://clinicaltrials.gov/ct2/show/NCT03587571	Operative vs. non-operative	Switzerland	18-65years (n=164)	Yes	OMAS	VAS-Foot and Ankle, AOFAS, FFI, Return to work, Kellgren-Lawrence Scale
Schweser (2019) Immediate Weight Bearing in Diabetic Ankle Fractures With Hindfoot Offloading (Diabetic Ankle) *NCT03966027* https://clinicaltrials.gov/ct2/show/NCT03966027	Post-operative management	USA	18+ years (n=25)	Yes	Adverse Events	AAOS and PROMIS Score
